# Thermo-Oxidative Aging Effects on Hyperelastic Behavior of EPDM Rubber: A Constitutive Modeling Approach

**DOI:** 10.3390/ma18102236

**Published:** 2025-05-12

**Authors:** Zhaonan Xie, Xicheng Huang, Kai Zhang, Shunping Yan, Junhong Chen, Ren He, Jiaxing Li, Weizhou Zhong

**Affiliations:** 1 Institute of Systems Engineering, China Academy of Engineering Physics, Mianyang 621999, China; 2 Shock and Vibration of Engineering Materials and Structures Key Laboratory of Sichuan Province, Mianyang 621999, China

**Keywords:** EPDM rubber, thermo-oxidative aging, constitutive model, hyperelastic behavior, Arrhenius relationship

## Abstract

The effect of thermo-oxidative aging on the hyperelastic behavior of ethylene propylene diene monomer (EPDM) rubber was investigated by a combined experimental and theoretical modeling approach. Firstly, the uniaxial tensile test of aged and unaged EPDM rubber was carried out. The test results show that the unaged EPDM rubber had the nonlinear large deformation characteristic of a “S” shape. The stiffness of the EPDM rubber was found to increase with the aging time and aging temperature. Then, in order to quantitatively characterize the hyperelastic behavior of unaged EPDM rubber, the fitting performances of the Mooney–Rivlin, Arruda–Boyce, and Ogden models were compared based on a uniaxial tensile stress–strain curve. The results show that the Ogden model provided a more accurate representation of the hyperelastic behavior of unaged EPDM rubber. Subsequently, the Dakin dynamic equation was adopted to associate the parameters of the Ogden model with the aging time, and the Arrhenius relationship was utilized to introduce the aging temperature into the rate term of the Dakin dynamic equation, thereby establishing an improved Ogden constitutive model. This improved model expanded the Ogden model’s ability to explain aging time and aging temperature. Finally, the improved model prediction results and the test results were compared, and they indicate that the proposed improved Ogden constitutive model can accurately describe the hyperelastic behavior of aged and unaged EPDM rubber.

## 1. Introduction

EPDM rubber is widely used in mechanical sealing [1,2], the tire industry [3], and vibration isolation [4] due to its excellent heat resistance, chemical stability, electrical insulation, radiation resistance, and mechanical properties. In these applications, it is often exposed to thermo-oxidative environments, where it inevitably reacts with oxygen, leading to its mechanical properties changing. Maintaining the long-term stability of its mechanical properties is crucial for the reliability of engineering systems. However, thermo-oxidative aging causes irreversible changes in the microstructure of rubber, such as the cross-linking of molecular chains, fracture, migration of additives, and the formation of oxidizing groups [5,6,7]. The changes generally reduce the mechanical properties of EPDM rubber and induce the long-term safety risk of engineering product. Therefore, investigating the effects of thermo-oxidative aging on the mechanical behavior of EPDM rubber is important as it plays a crucial role in understanding and predicting the long-term mechanical performance of it.

Hyperelasticity is a basic mechanical behavior of rubber, and it can describe its large deformation characteristics. Researchers have proposed many hyperelastic constitutive models, which can be classified into two categories: phenomenological models [8,9,10] and molecular chain network models [11,12]. Some reviews [13,14,15] have discussed the applicability, advantages, and limitations of these models, providing valuable guidance for model selection. Although existing hyperelastic constitutive models are generally sufficient to describe the deformation characteristics of rubber, the effects of thermo-oxidative aging are not considered in the models. It is difficult to describe the hyperelastic behavior of rubber with thermo-oxidative aging.

To further investigate the effects of thermo-oxidative aging on the hyperelastic behavior of rubber materials, researchers have explored various modeling approaches. The first modeling method is to decompose the strain energy into contributions from the original network and the newly formed network [16,17,18,19,20]. For example, Tobolsky et al. [21,22] proposed the dual-network model, which incorporates volume fraction change in both networks to account for the influence of thermo-oxidative aging. The second modeling method was proposed by Naumann [23], who developed a dynamic network model within the framework of the two-network theory, suggesting that thermo-oxidative aging involves a continuous process of molecular chain scission and re-crosslinking [24,25]. The third modeling method [26,27,28,29,30], based on finite deformation theory, decomposes the free energy function into three components: the volumetric term, original network term, and formed network term. And two internal state variables are introduced in these three terms to characterize the effects of thermo-oxidative aging, representing chain scission and crosslinking, respectively. Based on a strong thermo-chemical–mechanical coupling framework, a fourth modeling method [31,32,33] was provided. It can capture the chemical–physical evolution of the cross-linked network during the thermal aging process.

In addition to the above methods, there is another more commonly used modeling method. This method incorporates the factors of thermal oxidation aging into the parameters of the hyperelastic constitutive model and is suitable for rapid engineering assessment. For example, Li et al. [34,35] proposed a hyperelastic hybrid molecular chain model for viscoelastic damping materials subjected to thermo-oxidative aging. The model extends the elastic molecular chain density term by incorporating factors related to thermo-oxidative aging. Some researchers [36,37] have tried to relate chemical structure parameters to hyperelastic model parameters. The influence of thermo-oxidative aging on rubber mechanical behavior is reflected in the change in crosslinking density. Korba et al. [38] employed empirical formulations to characterize the changes in hyperelastic constitutive model parameters induced by thermo-oxidative aging. Some researchers [39,40,41,42] have combined empirical formulas related to chemical reactions, such as the Arrhenius relationship, to establish a hyperelastic constitutive model that considers the influence of thermo-oxidative aging.

This paper aims to establish a hyperelastic constitutive model for EPDM rubber that incorporates the coupled effects of aging temperature and aging time. Section 2 introduces the preparation of EPDM tensile specimens, the thermo-oxidative aging procedure, and the uniaxial tensile testing method. Section 3 analyzes the variations in the tensile stress–strain curves of both unaged and aged EPDM rubber. Section 4 proposes an improved Ogden model, which extends the Ogden model into an explicit aging temperature- and time-dependent model. The evaluation results show that the model can accurately describe the effects of the aging temperature and aging time on the hyperelastic behavior of EPDM rubber. Finally, Section 5 presents the conclusion.

## 2. Material and Methods

### 2.1. Material and Specimen

**Figure 1:**
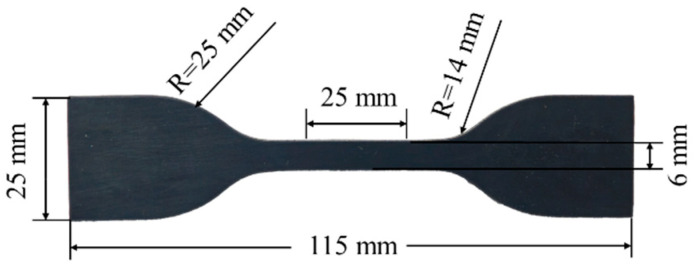
The EPDM rubber tensile specimen.

The EPDM rubber used in this study was made according to the work of Huang et al. [43], and the main components are shown in Table 1. The preparation method of EPDM rubber is as follows: EPDM, organo-modified montmorillonite (OMMT), compatibilizers, and other ingredients are added to a mixer. The mixing speed is 110 r/min, and the temperature is 90 °C for 15 min. Then, mixed rubber, ZnO, stearic acid, antioxidant, and other additives are sequentially added to a two-roll mill. The mixture, referred to as Compound I, is uniformly blended at 80 °C and then cooled to room temperature. Subsequently, vulcanizing agents and activators are incorporated into Compound I to produce Compound II. Finally, Compound II is vulcanized at 160 °C and 15 MPa for 1 h using a plate vulcanization machine, yielding an EPDM sheet with dimensions of 250 × 250 × 2 mm. The sheet is cut into dumbbell-shaped specimens following ISO 37-2024 [44] (specific dimensions are provided in Figure 1).

### 2.2. Test Methods

In order to investigate the coupling effects of the aging temperature and aging time on EPDM rubber, thermo-oxidative aging tests were conducted using various temperature–time combinations, as listed in Table 2. The samples were suspended in an air convection oven, and three parallel samples were set for each thermo-oxidative aging condition. According to ISO 37-2024 [44], if each test group has more than three sets of repeated specimens characterizing the uniaxial tensile behavior of rubber materials, it can ensure statistically valid results when studying the tensile response of rubber materials. After completing the aging process, the samples were removed from the oven and left in the air for more than 12 h. Uniaxial tensile tests were performed using a universal testing machine to evaluate the tensile response of EPDM rubber after thermo-oxidative aging. All of the tensile tests were carried out by displacement control mode until the tensile strain reached 200%. The tensile velocity was 500 mm/min during the tests. The strain data were recorded by the extensometer. The median of three repeated tests was taken as the stress–strain curve of the EPDM rubber.

## 3. Results

**Figure 2:**
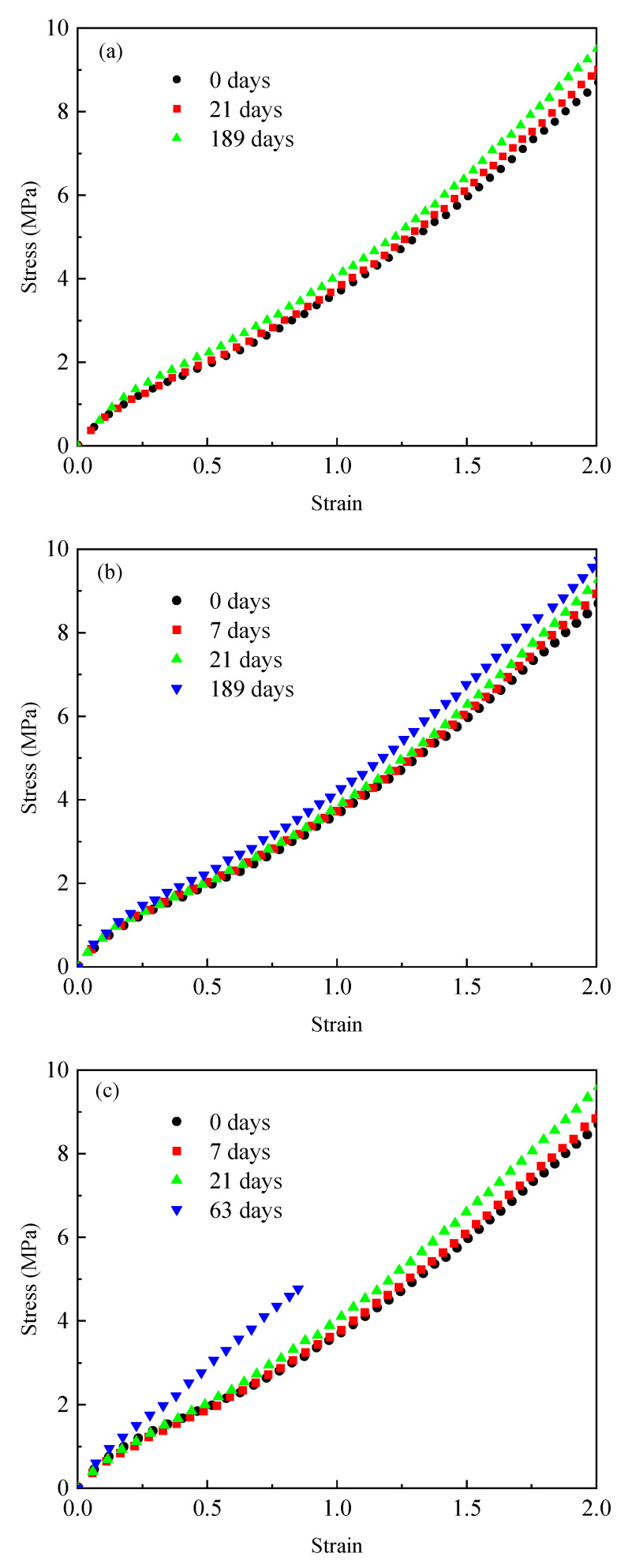
The tensile stress–strain curves of unaged EDPM rubber and those aged for different aging times at (**a**) 55 °C; (**b**) 80 °C; and (**c**) 120 °C.

Figure 2 shows the tensile stress–strain curves of EPDM rubber aged at 55 °C, 80 °C, and 120 °C, respectively. It can be found from Figure 2 that the stress value of unaged EPDM rubber will increase rapidly with an increase in the strain in the initial stage, which will increase slowly and then finally increase rapidly again after exceeding a certain strain value. This phenomenon is in line with the “S”-shaped nonlinear large deformation characteristics of EPDM rubber that has been established in the literature [45,46]. Furthermore, it can also be found that, with the extension of aging time, the slope of the stress–strain curve gradually increases. When the aging temperature was 55 °C and 80 °C for 189 days, the stress values of the EPDM rubber at 85% were 3.45 MPa and 3.53 MPa, respectively. When the aging temperature was 120 °C and the aging time was 63 days, the stress value of the EPDM rubber at 85% was 4.77 MPa, which was 27% higher than the stress value after aging at 55 °C for 189 days. These results indicate that, during the thermo-oxidative aging process, the stiffness of EPDM rubber increases with both aging time and aging temperature. This is consistent with the reported effects of aging temperature and time on the stiffness of EPDM rubber as established in the literature [39,47,48]. The reason for this change pattern is that increasing the aging temperature and time will accelerate the cross-linking reaction in rubber materials. This reaction will form additional chemical bonds between the molecular chains, resulting in an increase in the stiffness of the rubber. It should also be noted that, when the aging temperature is 120 °C and the aging time is 63 days, the stress–strain data were only collected at approximately 85%. This is because the excessive cross-linking reaction leads to a deterioration in the ductility of the rubber, causing it to break at approximately 85%. This phenomenon has also been found in the literature [42,47,49] for EPDM rubber. All these phenomena indicate that the aging temperature and aging time have a non-negligible influence on the mechanical properties of EPDM rubber.

## 4. Discussion

As shown in Section 3, the stress–strain curve of the EPDM rubber after thermo-oxidative aging showed two important characteristics: (1) unaged EPDM rubber presents an “S”-shaped nonlinear large deformation feature; and (2) an increase in aging time and aging temperature will lead to an increase in the stiffness of EPDM rubber. To accurately describe this behavior, this section introduces the establishment of the hyperelastic constitutive model of EPDM rubber and the expansion of model parameters regarding aging time and aging temperature.

### 4.1. Hyperelastic Constitutive Modeling

The mechanical properties of EPDM rubber can be described by the strain energy function *W*. In general, the strain energy function is expressed as a function *W*(***F***) of the deformation gradient tensor ***F***. The strain energy function is expressed as a function *W*(***U***) of the right tensile tensor ***U***. In a large deformation condition, the right Cauchy–Green tensor ***C*** = ***U***^2^ = ***F****^T^**F*** is an important physical quantity to describe the deformation of objects. Rubber is generally regarded as isotropic and a uniform hyperelastic material, so its strain energy function can be expressed as the *W*(*I*_1_,*I*_2_,*I*_3_) of the three invariants of the right Cauchy–Green tensor as follows:

(1) $$\left\{\begin{array}{l} I_{1}=\mathrm{tr}C=\lambda_{1}^{2}+\lambda_{2}^{2}+\lambda_{3}^{2}\\ I_{2}=\frac{1}{2}[(\mathrm{tr}C{)}^{2}-\mathrm{tr}C^{2})]=\lambda_{1}^{2}\lambda_{2}^{2}+\lambda_{2}^{2}\lambda_{3}^{2}+\lambda_{1}^{2}\lambda_{3}^{2}\\ I_{3}=\det C=\lambda_{1}^{2}\lambda_{2}^{2}\lambda_{3}^{2} \end{array}\right.,$$

where *λ*_1_, *λ*_2_, and *λ*_3_ are the three eigenvalues of the tensile tensor ***U***, which are physically interpreted as the three principal tensile ratios of the object.

Rubber usually also has incompressible properties, so *I*_3_ is equal to 1. For the principal direction of stress, *σ_i_* (*i* = 1,2,3) can be expressed as follows:

(2) $$\sigma_{i}=\frac{\partial W}{\partial \lambda_{i}}-p\lambda_{i}^{-1}=\frac{\partial W}{\partial I_{1}}\frac{\partial I_{1}}{\partial \lambda_{1}}+\frac{\partial W}{\partial I_{2}}\frac{\partial I_{2}}{\partial \lambda_{2}}-p\lambda_{i}^{-1},$$

where *σ_i_* (*i* = 1,2,3) is the nominal stress in the principal direction, and *p* is an arbitrary additive pressure associated with incompressible constraint condition.

For uniaxial tensile loading, the stress condition of *σ_2_* = *σ_3_* = 0 and the deformation characteristic of *λ*_1_ = *λ*, and *λ*_2_ = *λ*_3_ = *λ*^−1/2^ can be substituted into Equation (2), and the constitutive relationship under uniaxial tensile loading is obtained:

(3) $$\sigma_{1}=2(\frac{\partial W}{\partial I_{1}}+\frac{\partial W}{\partial I_{2}}\cdot \lambda^{-1})(\lambda -\lambda^{-2}).$$

At present, many strain energy functions are proposed to construct a reasonable strain energy function *W*. The strain energy function is with different complexity and scope of application. This section focuses on three classical models to determine an accurate model: the Mooney–Rivlin model, the Arruda–Boyce model, and the Ogden model.

#### 4.1.1. Mooney–Rivlin Model

The Mooney–Rivlin model [10] can effectively describe the hyperelastic constitutive relationship of small deformation and medium deformation, and its strain energy function is expressed as follows:

(4) $$W_{M-R}=C_{10}(I_{1}-3)+C_{01}(I_{2}-3),$$

where *C*_10_ and *C*_01_ are material parameters. When *C*_10_ + *C*_01_ > 0, the Mooney–Rivlin model remains stable.

By substituting Equation (4) into Equation (3), the constitutive relation of the Mooney–Rivlin model under uniaxial tensile load is obtained:

(5) $$\sigma_{1}=2(C_{10}+C_{01}\cdot \lambda^{-1})(\lambda -\lambda^{-2}).$$

#### 4.1.2. Arruda–Boyce Model

The Arruda–Boyce model [11] is a non-Gaussian chain statistical model that can effectively describe the hyperelastic relationship of large deformations under different deformation states. The strain energy function is expressed as follows:

(6) $$W_{A-B}=\mu_{0}\sum_{i=1}^{5}\frac{c_{i}}{N_{0}^{i-1}}(I_{1}^{i}-3^{i}),$$

where *c*_1_ = 1/2; *c*_2_ = 1/20; *c*_3_ = 11/1050; *c*_4_ = 19/7000; *c*_5_ = 519/673,750; *N*_0_ is the number of chain segments of the rubber molecular chain; *μ*_0_ is the rubber shear modulus and *μ*_0_ = *nkT*; *n* is the number of molecular chains per unit volume; *k* is the Boltzmann constant; and *T* is the absolute temperature.

By substituting Equation (6) into Equation (3), the constitutive relation of the Arruda–Boyce model under a uniaxial tensile load can be obtained:

(7) $$\begin{array}{c} \sigma_{1}=2\mu_{0}(\lambda -\lambda^{-2})[(\frac{1}{2}+\frac{1}{10N_{0}}(\lambda^{2}+2\lambda^{-1})+\frac{11}{350N_{0}^{2}}{\left(\lambda^{2}+2\lambda^{-1}\right)}^{2}+\\ \;\frac{19}{1750N_{0}^{3}}{\left(\lambda^{2}+2\lambda^{-1}\right)}^{3}+\frac{519}{134750N_{0}^{4}}{\left(\lambda^{2}+2\lambda^{-1}\right)}^{4})] \end{array}.$$

#### 4.1.3. Ogden Model

Based on the Valanis–Landel hypothesis [50], the Ogden model [9] takes the principal elongation ratio *λ_i_* as the independent variable. This model effectively describes the full-range mechanical response of rubber-like materials under multiaxial stress. The strain energy function is expressed as follows:

(8) $$W=\sum_{i=1}^{N}\frac{\mu_{i}}{\alpha_{i}}(\lambda_{1}^{\alpha_{i}}+\lambda_{2}^{\alpha_{i}}+\lambda_{3}^{\alpha_{i}}-3),$$

where *α_i_* and *μ_i_* are the material parameters; *N* is the order of the polynomial; and *α_i_* and *μ_i_* satisfy the relation $\mu =\sum_{i=1}^{n}\mu_{i}\alpha_{i}/2$, where *μ* represents the initial shear modulus (which must be positive) [9,51]. In order to improve the analysis accuracy of the Ogden model and ensure the convergence of finite element calculations, the order of *N* is generally less than 4. For improving the stability of the model, it is necessary to set the constraints $\sum_{i=1}^{n}\mu_{i}\alpha_{i}\text{>}0$.

By substituting Equation (8) into Equation (3), the constitutive relation of the Ogden model under a uniaxial tensile load can be obtained:

(9) $$\sigma_{1}=\sum_{i=1}^{N}\mu_{i}(\lambda^{\alpha_{i}-1}-\lambda^{-\frac{1}{2}\alpha_{i}-1}).$$

To compare with the Mooney–Rivlin model and Ogden model under the same number of parameters, *N* was set as 1 in this paper.

#### 4.1.4. Model Comparison

**Figure 3:**
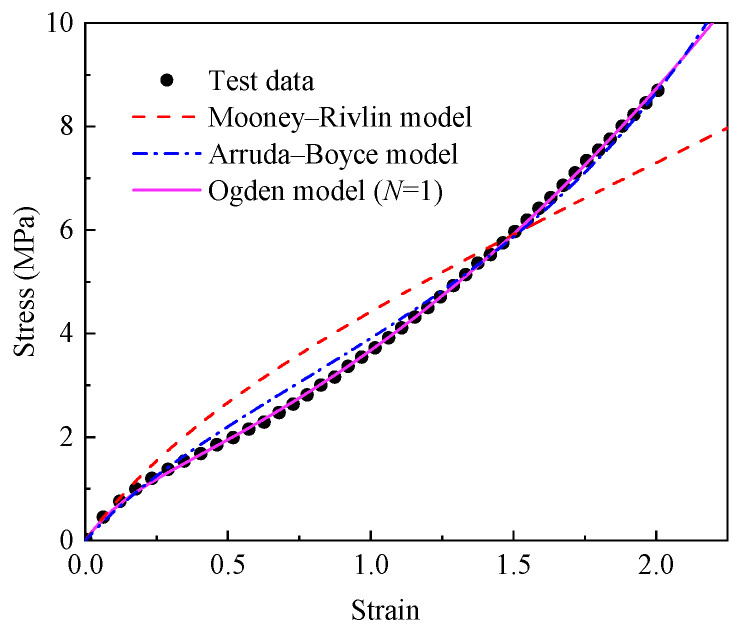
The uniaxial tensile stress–strain fitting curves of unaged EPDM rubber based on the Mooney–Rivlin model, Arruda–Boyce model, and Ogden model (*N* = 1).

The parameters of the Mooney–Rivlin, Arruda–Boyce, and Ogden models were obtained by fitting the experimental data using the nonlinear least squares method, the fitting results of which are shown in Figure 3. It can be observed from Figure 3 that the Mooney–Rivlin model failed to capture the S-shaped stress–strain behavior of the EPDM rubber, whereas both the Arruda–Boyce and Ogden models provided a satisfactory representation of this characteristic. To more accurately evaluate the reliability of the model’s predictions, multiple evaluation metrics should be employed rather than relying on a single one [52]. Here, the determination coefficient (R^2^), root mean square error (RMSE), and the mean absolute percentage error (MAPE) were adopted to comprehensively evaluate the predictive performance of the Mooney–Rivlin model, the Arruda–Boyce model, and the Ogden model for the tensile stress–strain curve of unaged EPDM rubber.

R^2^ can reflect the model’s ability to explain the trend of data variation, which is calculated by the residual sum of squares (RSS) and the total sum of squares (TSS):

(10) $$\left\{\begin{array}{l} R^{2}=1-\mathrm{RSS}/\mathrm{TSS}\\ \mathrm{TSS}=\sum_{i=1}^{n}{\left(\sigma_{\mathrm{test}}-{\bar{\sigma }}_{\mathrm{test}}\right)}^{2}\\ \mathrm{RSS}=\sum_{i=1}^{n}{\left(\sigma_{\mathrm{prediction}}-\sigma_{\mathrm{test}}\right)}^{2} \end{array}\right.,$$

where $\sigma_{\mathrm{test}}$ is the test value, ${\bar{\sigma }}_{\mathrm{test}}$ is average of the test values, $\sigma_{\mathrm{prediction}}$ is the model prediction value, and *n* is the number of test data points. The closer the value of R^2^ is to 1, the stronger the model’s ability to explain the variation in the data.

RMSE can reflect the influence of large absolute errors, and its expression its expression is given by the following:

(11) $$\mathrm{RMSE}=\sqrt{\frac{1}{n}\sum_{i=1}^{n}{\left(\sigma_{\mathrm{test}}-\sigma_{\mathrm{predicton}}\right)}^{2}}.$$

The smaller the RMSE value is, the better the handling of the outliers of the model is, and the smaller the extreme value of the prediction deviation is.

MAPE can compare the data of different magnitudes, and its expression is as follows:

(12) $$\mathrm{MAPE}=\frac{100\%}{n}\sum_{i=1}^{n}\left|\frac{\sigma_{\mathrm{test}}-\sigma_{\mathrm{prediction}}}{\sigma_{\mathrm{test}}}\right|.$$

The closer the MAPE value is to zero, the smaller the relative deviation between the model’s prediction results and the test results will be and the higher the prediction reliability will also be.

Table 3 presents the values of the R^2^, RMSE, and MAPE for the Mooney–Rivlin model, the Arruda–Boyce model, and the Ogden model. It can be found from Table 3 that the Mooney–Rivlin model, whether it was the R^2^, RMSE, or MAPE, was significantly inferior to the other two types of models. Both the Arruda–Boyce model and the Ogden model had very high R^2^ values. However, the RMSE of the Ogden model was 0.0331, which was 74.4% lower than that of the Arruda–Boyce model, and the MAPE was 1.5281, which was 67.57% lower than that of the Arruda–Boyce model. The above results indicate that the Ogden model can describe the stress–strain curve of unaged EPDM rubber more accurately. Therefore, the Ogden model was selected to describe the hyperelastic constitutive model for the EPDM rubber, with the fitted parameters *μ*_0_ = −0.8414 and *α*_0_ = −6.26.

### 4.2. Effect of the Temperature and Aging Time on the Model Parameters

Based on Equation (9), the different material parameters *μ* and *α* can be obtained at various aging temperatures and times. It implies that the Ogden model has implicitly taken into account the effects of aging temperature and time. However, it is difficult to characterize the hyperelastic behavior of EPDM rubber at different aging temperatures and aging times by uniform parameters. In order to facilitate engineering applications, it is necessary to establish the relationship of the material parameters of the Ogden model and the aging temperature and aging time, as well as extend it into an explicit correlation form of the aging temperature and aging time.

The Ogden model is used to fit the stress–strain data at different aging times and temperatures. The corresponding material parameters *μ* and *α* were obtained, as shown in Table 4. It can be seen from Table 4 that the parameter *μ* gradually increased with the aging temperature and aging time, while the parameter *α* presented an opposite trend.

For the thermo-oxidative aging process of rubber, the thermo-oxidative aging rate can be expressed by a Dakin-type kinetic relation [39,42,53]:

(13) $$\frac{dq}{dt}=k(T)f(q),$$

where *q* is the investigated property, *t* is the thermo-oxidative aging time, *k*(*T*) is the kinetic rate related to the absolute temperature *T* (K), and *f*(*x*) is a function of the degree of thermo-oxidative aging. The kinetic rate *k*(*T*) is assumed to follow the Arrhenius relation:

(14) $$k(T)=A\exp\left(-\frac{E_{a}}{\mathrm{RT}}\right),$$

where *A* is the pre-exponential factor, *E_a_* is the activation energy, and *R* is the gas constant (8.314 J/(K·mol)).

**Figure 4:**
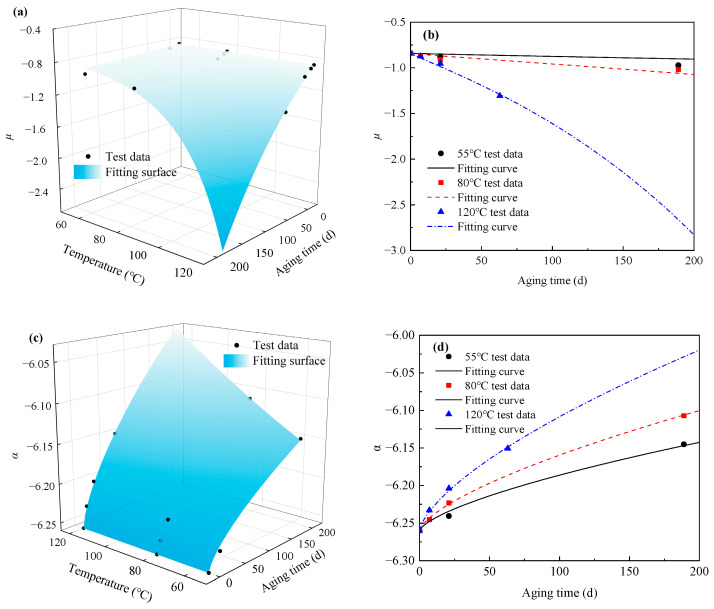
The variation of parameters *μ* and *α* with different aging temperatures and aging times is expressed in (**a**,**c**) three dimensions and (**b**,**d**) two dimensions.

Equation (13) is usually used to describe the influence of thermo-oxidative aging on its mechanical properties, and the material parameters *μ* and *α* contribute to the tensile modulus, so the changes in the two parameters due to thermo-oxidative aging can be described by Equation (13). Figure 4 show the changes in *μ* and *α* with the aging time and aging temperature. It can be found that *μ* and *α* at different temperatures show an exponential change trend with the time increasing. Therefore, *f*(*q*) can be defined as follows:

(15) f(q)=q, (q=μ,α).

Equation (13) was substituted by Equations (14) and (15), and the differential Equation (13) was then solved. As such, the relationship between parameters *μ* and *α* with the aging temperature and aging time could be obtained:

(16) $$q(t,T)=q_{0}\exp\left[\mathrm{At}\exp\left(-\frac{E_{a}}{\mathrm{RT}}\right)\right],$$

where *q*_0_ is the material parameter of unaged EPDM rubber. Equation (16) can be fitted to obtain the thermo-oxidative aging parameter *A* by the nonlinear least square method, but it was found that the fitting result was not ideal. In order to solve this problem, an improved form of Equation (16) was proposed, the specific expression is as follows:

(17) $$q(t,T)=q_{0}\exp\left[At^{n}\exp\left(-\frac{E_{a}}{\mathrm{RT}}\right)\right],$$

where *n* is a positive real number, providing additional freedom [40] to characterize the thermo-oxidative aging material parameters *μ* and *α*.

Combined with the experimental data, the nonlinear least square method was used to fit the parameters *μ* and *α*, and the fitting results are shown in Figure 4a,c. In order to improve the fitting effect, the fitting results of parameters *μ* and *α* were represented in two dimensions, as shown in Figure 4b,d. Table 5 presents the values of the R^2^, RMSE, and MAPE values for parameter *μ* and *α*. A high R^2^ value, a low RMSE value, and a low MAPE value indicates that Equation (17) can describe the changes in parameters *μ* and *α* with the aging time and aging temperature. The fitting results obtained are as follows:

(18) $$\left\{\begin{array}{l} \mu =-0.8414\exp\left[15019.628\cdot t^{0.901}\cdot \exp(\frac{-5579.926}{T})\right]\\ \alpha =-6.26\exp\left[-0.04223\cdot t^{0.6758}\cdot \exp(-\frac{1438.051}{T})\right] \end{array}\right..$$

Substituting Equation (18) into Equation (9), the hyperelastic constitutive model of EPDM rubber when considering thermo-oxidative aging was established. The model requires eight parameters, and the number of parameters in this model is common in studies [38,39,40,42] related to thermo-oxidative aging hyperelastic constitutive models.

### 4.3. Model Verification

**Figure 5:**
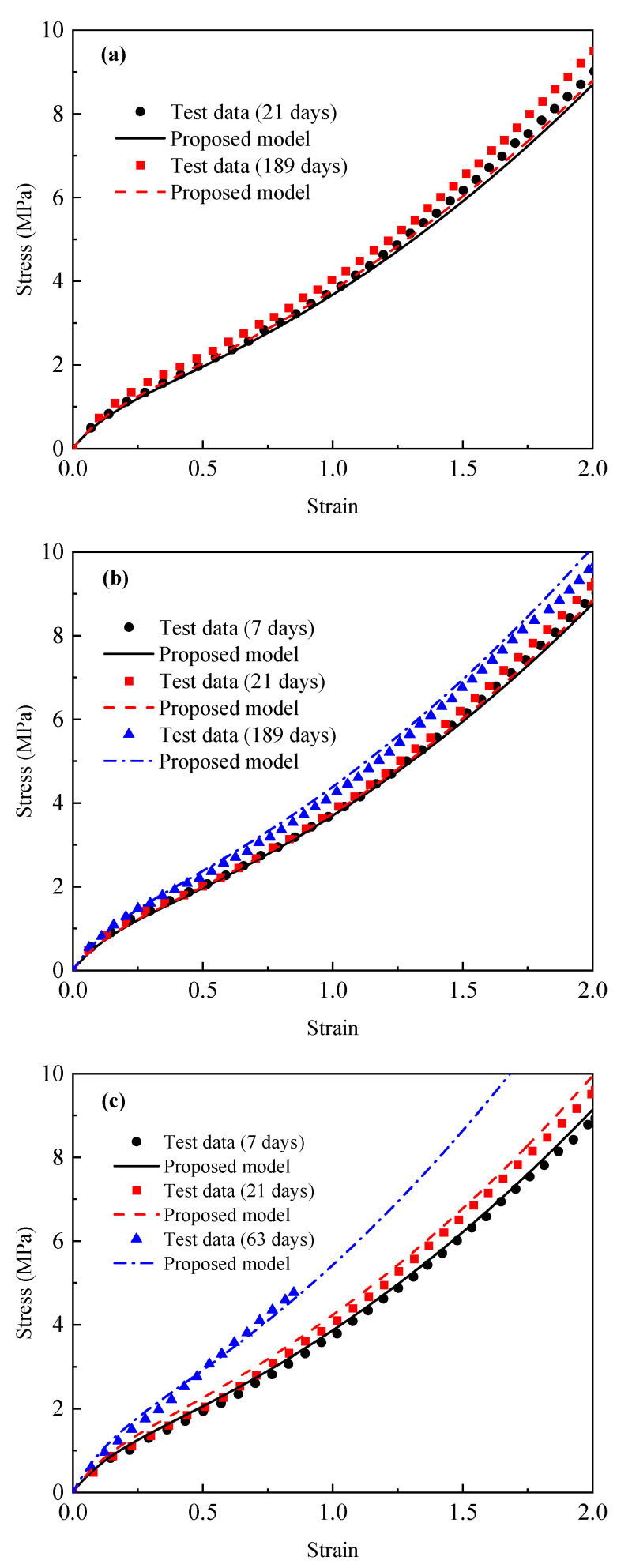
Comparisons between the prediction results of the proposed model and the stress–strain test data of the EPDM aged at (**a**) 55 °C, (**b**) 80 °C, and (**c**) 120 °C for different times.

To validate the accuracy of the established constitutive model, a comparison between the experimental results and the prediction results of the proposed model was conducted, as shown in Figure 5. It can be seen that the proposed model can effectively predict the experimental results at 55 °C for 21 days; 80 °C for 7, 21, and 189 days; and 120 °C for 7, 21, and 63 days. The prediction results of the aging at 55 °C for 189 days were slightly different from the experimental data. In order to further evaluate the proposed model prediction effect, the R^2^, RMSE and MAPE values were used for quantitative description.

The R^2^, RMSE, and MAPE values between the prediction results are shown in Table 6. It shows that the proposed model achieved an R^2^ value that was consistently above 0.97, an RMSE value below 0.4 MPa, and a MAPE value under 8%, which means that the proposed model can describe the behavior of aged EPDM rubber well.

**Figure 6:**
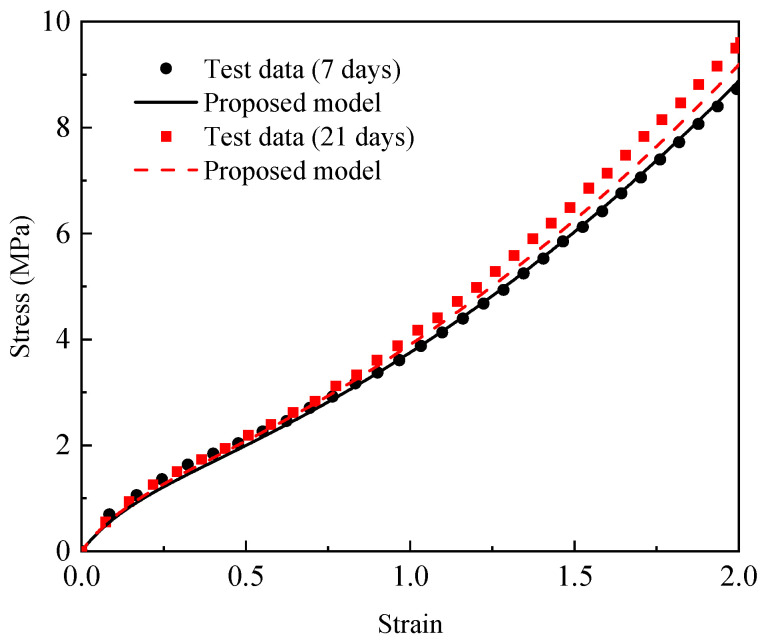
Comparisons of the prediction results of the proposed model and the stress–strain test data of EPDM aged at 100 °C for 7 days and 21 days.

To further validate the rationality of the model, two additional thermo-oxidative aging tensile tests were conducted. The same batch of EPDM samples were used in the two tests, and the aging conditions were 100 °C aging for 7 days and 21 days, respectively. The tensile test data of the aging at 100 °C for 7 days and 21 days were calculated using the proposed model, the results of which are shown in Figure 6. It can be observed that the predicted stress–strain curves exhibited minor deviations from the experimental data. These deviations can be quantitatively evaluated using the R^2^, RMSE, and MAPE. For the EPDM tensile data conditions of 100 °C aging temperature and 7 days aging time, the R^2^ value, RMSE value, and MAPE value of the proposed model were 0.998, 0.0833 MPa, and 3.2738, respectively. For the EPDM tensile data conditions of the 100 °C aging temperature and 21 days aging time, the R^2^ value, RMSE value, and MAPE value of the proposed model were 0.991, 0.2525 MPa, and 4.6278, respectively. These results show that the proposed model can accurately describe the mechanical response of EPDM rubber under different aging temperatures and aging times.

## 5. Conclusions

This study carried out experimental and theoretical model research on the coupling effect of the aging temperature and aging time on the hyperelastic behavior of EPDM. Firstly, uniaxial tensile tests were performed on both unaged and aged EPDM rubber to investigate the effects of the aging temperature and aging time on its hyperelastic behavior. Secondly, based on the test results, an improved Ogden constitutive model was proposed by extending the parameters of the Ogden model to explain the aging temperature and aging time. Finally, by comparing the prediction results of the model with the experimental results, the validity of the model was proved. The main conclusions drawn from this study include the following:

- The tensile stress–strain curve of EPDM rubber exhibits a typical S-shape large deformation characteristic. EPDM rubber gradually hardens when the aging time and aging temperature increases.
- The fitting performance of the Mooney–Rivlin, Arruda–Boyce, and Ogden models was evaluated using the R^2^, MAPE, and RMSE. The results show that the Ogden model more accurately describes the hyperelastic behavior of unaged EPDM rubber.
- An improved Ogden model was proposed. This model extends the aging time correlation of the parameters through the Dakin dynamic relationship and the aging temperature correlation of parameters through the Arrhenius relationship.
- In the strain range of 200%, the proposed model was able to capture the hyperelastic behavior of EPDM rubber when considering the influence of thermo-oxidative aging well.

Overall, this study provides an improved Ogden model to simulate the hyperelastic behavior of both unaged and aged EPDM rubber. This model can also be applied to describe the thermo-oxidative aging hyperelastic behavior of other rubber-like materials. Future research could focus on extending the proposed method to more complex loading conditions or different types of rubber-like materials to further expand its applicability in the prediction of long-term mechanical behavior.

## Figures and Tables

**Table 1:** Formulation of the EDPM compounds.

Ingredients	Value [phr]
EPDM	100
Carbon black	30
Organo-modified montmorillonite	25
Compatibilizers	10
Zinc oxide	5
Antioxidant	3
Vulcanizator	3

**Table 2:** The specimen thermo-oxidative aging test scheme.

Aging Temperature (°C)	Aging Time (Days)			
55 °C	0	21	189	/
80 °C	0	7	21	189
120 °C	0	7	21	63

**Table 3:** The values of the R^2^, RMSE, and MAPE values corresponding with the Mooney–Rivlin model, Arruda–Boyce model, and Ogden model.

Model	R^2^	RMSE (MPa)	MAPE (%)
Mooney–Rivlin	0.9229	0.6668	17.2422
Arruda–Boyce	0.9971	0.1293	4.7118
Ogden (*N* = 1)	0.9998	0.0331	1.5281

**Table 4:** The fitting parameters of the Ogden model (*N* = 1) to the test data of the EPDM rubber at different aging temperatures and times.

Aging Temperature (°C)	Aging Time (d)	*μ*	*α*
55	21	−0.88	−6.24
	189	−0.972	−6.145
80	7	−0.87	−6.243
	21	−0.9122	−6.223
	189	−1.021	−6.107
120	7	−0.875	−6.233
	21	−0.9545	−6.209
	63	−1.308	−6.151

**Table 5:** The values of R^2^, RMSE, and MAPE corresponding to the parameters *μ* and *α*.

Parameter	R^2^	RMSE	MAPE (%)
*μ*	0.9976	0.0025	0.0286
*α*	0.9397	0.032	2.5557

**Table 6:** The values of the R^2^, RMSE, and MAPE corresponding to the improved Ogden model under different aging temperatures and different aging times.

Aging Temperature (°C)	Aging Time (d)	R^2^	RMSE (MPa)	MAPE (%)
55 °C	21	0.9941	0.1908	3.7157
	189	0.9769	0.3869	7.9223
80 °C	7	0.9977	0.1176	3.3618
	21	0.9941	0.1969	4.2133
	189	0.9937	0.2110	3.7725
120 °C	7	0.9974	0.1278	3.9492
	21	0.9936	0.2143	6.4662
	63	0.9895	0.1347	7.2237

## Data Availability

The original contributions presented in the study are included in the article; further inquiries can be directed to the corresponding author.
